# A New Vegetation Segmentation Approach for Cropped Fields Based on Threshold Detection from Hue Histograms

**DOI:** 10.3390/s18041253

**Published:** 2018-04-18

**Authors:** Mohamed Hassanein, Zahra Lari, Naser El-Sheimy

**Affiliations:** 1 Department of Geomatics Engineering, University of Calgary, 2500 University Dr NW, Calgary, AB T2N1N4, Canada; elsheimy@ucalgary.ca; 2 Leica Geosystems Ltd.; 245 Aero Way NE, Calgary, AB T2E6K2, Canada; zahra.lari@leica-geosystems.com

**Keywords:** vegetation segmentation, threshold detection, hue histogram, UAV, precision agriculture

## Abstract

Over the last decade, the use of unmanned aerial vehicle (UAV) technology has evolved significantly in different applications as it provides a special platform capable of combining the benefits of terrestrial and aerial remote sensing. Therefore, such technology has been established as an important source of data collection for different precision agriculture (PA) applications such as crop health monitoring and weed management. Generally, these PA applications depend on performing a vegetation segmentation process as an initial step, which aims to detect the vegetation objects in collected agriculture fields’ images. The main result of the vegetation segmentation process is a binary image, where vegetations are presented in white color and the remaining objects are presented in black. Such process could easily be performed using different vegetation indexes derived from multispectral imagery. Recently, to expand the use of UAV imagery systems for PA applications, it was important to reduce the cost of such systems through using low-cost RGB cameras Thus, developing vegetation segmentation techniques for RGB images is a challenging problem. The proposed paper introduces a new vegetation segmentation methodology for low-cost UAV RGB images, which depends on using Hue color channel. The proposed methodology follows the assumption that the colors in any agriculture field image can be distributed into vegetation and non-vegetations colors. Therefore, four main steps are developed to detect five different threshold values using the hue histogram of the RGB image, these thresholds are capable to discriminate the dominant color, either vegetation or non-vegetation, within the agriculture field image. The achieved results for implementing the proposed methodology showed its ability to generate accurate and stable vegetation segmentation performance with mean accuracy equal to 87.29% and standard deviation as 12.5%.

## 1. Introduction

The agriculture industry is a major source of livelihood in the whole world. Its importance has been enhanced by its role to supply food, clothing, medicine, and employment opportunities to all human beings. Agriculture has an important ability to enhance the environment condition through providing different biological products [1]. Due to such importance and roles, it was vital to transform the agriculture industry into a knowledge-based industry to enhance the benefits of its outputs. Therefore, a major step is to apply a management system to the agriculture industry which can achieve an economically and an environmentally development for the agriculture process. Meanwhile, it is important to avoid the side effects of using the different agricultural inputs such as water, chemical herbicides, fertilizers, and machine fuel, on the surroundings. For such needs, a special management system was needed to distribute the different inputs based on the needs of each spot in the agriculture field to avoid any misuse of the resources and thus avoid any harm effects on the surrounding environment. Such management system was named as smart farming or precision agriculture (PA), which is defined as using the right technology to perform the right agriculture activity at the right time and the right location [2].

Through the last three decades the use of PA as a management system for the agriculture industry has become one of the top ten revolutions in agriculture [3]. Generally, PA performs the distribution of the inputs in a customized manner based on the needs of each part of the field. Therefore, a PA management system depends on using different technologies to collect suitable information and detect the needs of each zone in the field along with controlling the distribution process of these inputs. A leading example for these technologies is remote sensing (RS) which has a huge ability to provide valuable information for PA activities. Common platforms such as satellites and airplanes provide RS technology with the ability to acquire images covering large areas within short time [4]. Such collected images are used to monitor the crop growth, crop stress, and predict crop yield. Despite such valuable information that space-borne and aerial-borne remote sensing systems can provide, there are shortcomings with these systems that affect the use of RS technology for PA, which can be showed by comparing the use of GNSS technology with RS technology for PA. Different surveying reports showed that the application of GNSS technology for PA is more widely practiced compared to the use of RS technology [5].

Generally, space-borne and airborne RS systems are limited by the low temporal and spatial resolution compared to the needs of farmers [6]. On the other hand, terrestrial RS systems are capable to increase the spatial resolution along with controlling the temporal resolution [7]. Though, such systems have other limitations especially with the needed time to cover the large agriculture fields. Therefore, unmanned aerial vehicles (UAVs) have been introduced and utilized as an alternative solution to fit the gaps between aerial and terrestrial RS systems.

Through the last few decades, UAVs have showed a great potential as an imagery system platform that can be used for different applications [8]. The UAV imagery systems are considered as a low-cost alternative for regular remote sensing systems such as aerial and satellite-based systems. They are providing higher spatial resolution as they are flying at lower altitudes, where such spatial resolution is capable to provide the needed information for different agriculture applications as weed detection and crop health monitoring. Moreover, UAV systems can provide high temporal resolution imaging systems which are capable of real-time/flexible imagery acquisition [9,10,11,12,13]. Therefore, UAV imagery system has been implemented for different PA applications. These applications include crop health monitoring [14], weed management [15,16], and crop row detection [17].

The aforementioned PA applications mainly depend on using computer vision or image processing approaches to extract the suitable information to achieve the targeted goals of these applications. Generally, for the mentioned applications, vegetation extraction, or vegetation segmentation process is considered as an initial step to achieve the objective of each application [7,16,18,19]. The vegetation extraction process aims to classify the different pixels in the acquired images into two classes which are vegetation class and non-vegetation class. The vegetation class includes all the plant objects in the images as crops and weeds, while the remaining objects belong to the non-vegetation class. To avoid any misclassification that can affect the quality of the targeted application, the vegetation extraction, vegetation segmentation, or background removal process should be performed in an appropriate way. Usually, the vegetation extraction can be easily performed using the vegetation indexes to detect the vegetation objects. These vegetation indexes such as NDVI, Normalized Red (NR), Normalized Green (NG), Difference Vegetation Index (DVI), and many more, are applied using multispectral images, taking the advantage of having different spectral channels especially the NIR, R, and G channels [2].

Unfortunately, the use of multispectral cameras for the UAV imagery systems is considered as a disadvantage due to the high cost of these sensors compared to the low-cost off-shelf RGB cameras. Such, cost is considered as a major obstacle of expanding the use of UAV for PA applications [4]. Thus, there was a need to develop the ability of performing the vegetation extraction process using RGB images. Therefore, different vegetation extraction methods were developed to be applied on RGB images. These methods can be classified into two general approaches which are: the combination of color index-based and threshold index-based approaches and the machine learning-based approaches [19].

### 1.1. Color Index-Based Segmentation

In computer vision and image processing applications, color is considered as an important factor due to its ability to discriminate between objects. So, several researchers used color characteristics to distinguish green vegetation objects from soil or background for different agriculture applications. Generally, the color index-based approaches convert the RGB values into modified grayscale using a color index. Then, the generated grayscale image is used to perform the vegetation extraction process using the suitable threshold value. Such combination is preferred for two main advantages: the simplicity of implementation, along with the high computational efficiency. These advantages are crucial for any real or near real-time agriculture application, which is a needed characteristic for any system provided for farmers.

Generally, within any RGB color space image, the color of each pixel is represented using the combination of three values which are the R, G, and B. Therefore, to make a transformation of the pixel color from the three values of R, G, and B into one grayscale value while keeping the ability of discrimination the vegetation object, is a challenging procedure. Thus, different researchers developed multiple color index-based techniques that generate different RGB vegetation indexes to create the modified grayscale images. These vegetation indexes are using different formulas or equations by changing the weights of the R, G, or B values as shown through Equations (1)–(4):

(1) $$\mathrm{NDI}=128\times \left(\left(\frac{G-R}{G+R}\right)+1\right)$$

(2) ExG=2×G−R−B

(3) ExR=1.3×R−G

(4) CIVE=0.441×R−0.811×G+0.385×B+18.78745

Thus, the modified grayscale images can provide the ability to discriminate the vegetation objects as they have different grayscale values from other objects. Such an approach was followed by different researchers for proposing different vegetation extraction indexes such as the Normalized Difference Index (NDI) [20], the Excess Green Index (ExG) [21], the Excess Red Index (ExR) [22], and the Color Index of Vegetation Extraction (CIVE) [23]. While other researchers worked by using a combination of different vegetation indexes to create a modified one. Examples for such techniques can be shown in Excess Green minus Excess Red Index (ExGR) [24], Combined Indices 1 (COM1) [25], and Combined Indices 2 (COM2) [26], as shown in Equations (5)–(7):

(5) ExGR=ExG−ExR

(6) COM1=ExG+CIVE+ExGR+VEG

(7) COM2=0.36×ExG+0.47×CIVE+0.17×VEG

Finally, it is important to note that color index-based techniques need a threshold value to convert the modified grayscale image into a vegetation binary image, where the vegetation objects are white while the remaining objects are black.

### 1.2. Threshold Index-Based Segmentation

As mentioned, the color index-based approaches need a threshold value to generate the vegetation binary image as the main output of the vegetation segmentation process. Therefore, different threshold detection approaches were proposed for such purpose. Among those techniques, Otsu threshold [27] is considered as one of the leading thresholding techniques. Otsu’s method is implemented in many segmentation applications. Therefore, many researchers used it for the vegetation segmentation process [28,29]. Though, due to the importance of the threshold value for the binarization process, different researches were developed for producing other threshold detectors.

For example, a suitable threshold value was introduced through calculating the local homogeneity value of the grayscale image. Different researchers introduced an algorithm for vegetation segmentation through converting the RGB images into grayscale then the method generates local homogeneity images to detect a homogeneity threshold [30,31]. The authors empirically detect a threshold value as 0.97 to be used for vegetation segmentation process. Moreover, the histogram entropy was used to produce a threshold detection method to be applied for weighted grayscale image [32]. Later another threshold detection method was introduced based on the assumption of existing two Gaussian distributions for the existing colors in the image [33]. The authors assumed that the highest mean represents the vegetation distribution while the lower mean represents the non-vegetation distribution. A similar threshold detection methodology was also introduced later, where the authors assumed that there are two groups of pixels and the threshold value is the one that minimizes the variances sum of the two groups [34].

The main limitation of the different proposed threshold techniques is the stability of the binarization accuracy of the system, as any mis-segmentation is generally caused by the error of the detected threshold. So, if the detected threshold is not appropriately estimated, the generated segmentation process will be highly affected. Moreover, different threshold-based methodologies follow the assumption of the equal presence of vegetation and non-vegetation objects in the collected images. Such assumption is not totally valid through the different growth stages. On the other side, color index-based techniques that convert the RGB values into grayscale don’t always result in a good discriminative grayscale image. This is mainly due to the effect of illumination conditions over the RGB values. Therefore, although the simplicity and the developed techniques offered by color index-based and threshold index-based approaches, they have some limitations as the effect of illumination changing conditions on the achieved vegetation segmentation results, particularly in sunny and overcast conditions, so there was a need for more research to investigate other alternatives, avoid these limitations, and achieve better results.

To avoid the illumination effects, the use of different color spaces such as HSV or LAB instead of RGB was considered. The main motivation of using these color spaces is mainly the ability to describe the object color away from the illumination of the object’s color. For more explanation, the RGB color space describes the object color using a combination of three values which are the R, G, and B. The combination of the three values describe the color content along with the illumination content without discriminating these two contents. On the other hand, HSV color space describe the object’s color content discriminated from the illumination content. HSV color space is composed of three channels which are the hue, saturation, and vibrancy, or value. The hue channel represents the color of the object, while the other two channels, S and V, describe the illumination content of the object. So, the H value provides a separate description of the object color rather than the illumination content. Hence, the proposed vegetation segmentation technique adopted the use of HSV as the color space of the images.

Generally, the proposed approach adopts a main assumption that any image for an agriculture field contains two main colors, or classes. The first class is the vegetation objects with green color, while the second class is the background soil of the field with yellow or brown color. Therefore, the proposed paper proposes a vegetation segmentation methodology following the threshold index-based approaches to discriminate between these two main classes or colors. In the following section, the proposed methodology will be discussed. Then, a description for the data acquisition process will be provided. The following section provides detailed steps to test the proposed vegetation segmentation approach. Later, an analysis of the achieved results will be presented. Finally, the paper’s main conclusions will be presented. 

## 2. Methodology

As discussed in previous sections, the vegetation segmentation process is an initial and vital step for different agriculture applications such as weed detection and crop row detection, especially, when working with RGB images. Due to the importance of this step, different solutions and approaches have been proposed to perform the vegetation segmentation as accurate and fast as possible. The main motivation for targeting higher accuracy vegetation segmentation process is to enhance the targeted agriculture application results. On the other hand, the fast performance is essential due to the importance of time for the different PA applications. The proposed vegetation segmentation technique targets the RGB images, as they are the main output from low-cost UAV imagery systems. The proposed methodology follows the procedure of threshold index-based approaches to achieve the simplicity of implementation and the low processing time.

**Figure 1:**
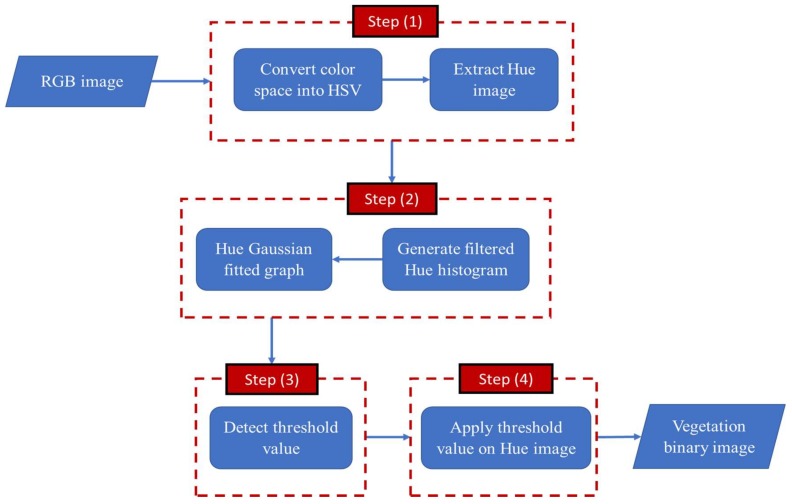
Main steps of the proposed methodology.

As shown in Figure 1, the proposed vegetation segmentation technique has four main steps. First, the acquired image’s color space is converted from RGB into HSV. Then, a filtered Hue histogram graph for each image is generated, along with a second graph which represents the Gaussian curve that fits the hue histogram. These two graphs are used in the threshold detection process in the third step. Finally, the detected threshold value is applied on the hue grayscale image to generate the vegetation binary image. Through the following subsections, a full description of the proposed technique is introduced.

### 2.1. HSV Color Space

Generally, the acquired images from a low-cost UAV imagery system are RGB images, where the color of any object in these images is represented with the combination values of R, G, and B channels. The main problem with such color representation is the impact of changes in illumination conditions on the objects’ colors. So, there was a need to use a different color space that describes the objects’ colors independent of the illumination effect.

For such needs, the HSV as a color space of the images could be used instead of the RGB. The main difference between these color spaces is mainly the color representation. The HSV color space represents the object’s color using three different parameters which are the hue (H), saturation (S), and value (V). The H represents the color of the object while the S and V values represent the illuminance state of the object’s color. Such description provides the ability to discriminate the color from the illuminance and therefore avoids the effect of illumination changes on the object color. Therefore, the proposed vegetation segmentation technique adopts the use of HSV color space.

Hence, as shown in Figure 1, the first step of the proposed methodology is to convert the image’s color space from the RGB into HSV. Generally, the transformation process of the pixel’s color value from RGB into HSV can be performed using the following equations [35]:

(8) $$R^{\prime }=\frac{R}{255}\;\&\;G^{\prime }=\frac{G}{255}\;\&\;B^{\prime }=\frac{B}{255}$$

(9) $$M=\max\left(R^{\prime },G^{\prime },B^{\prime }\right)\;\&\;m=\min\left(R^{\prime },G^{\prime },B^{\prime }\right)\;\&\;C=M-m$$

(10) $$H=\{\begin{array}{cc} 0^{0} & \mathrm{if}\;C=0\\ 60^{{}^{\circ}}\times \left(\frac{G^{\prime }-B^{\prime }}{C}\mathrm{mod}6\right) & \mathrm{if}\;M=R^{\prime }\\ 60^{{}^{\circ}}\times \left(\frac{B^{\prime }-R^{\prime }}{C}+2\right) & \mathrm{if}\;M=G^{\prime }\\ 60^{{}^{\circ}}\times \left(\frac{R^{\prime }-G^{\prime }}{C}+4\right) & \mathrm{if}\;M=B^{\prime } \end{array}$$

(11) $$S=\{\begin{array}{cc} 0 & \mathrm{if}\;M=0\\ \frac{C}{M} & \mathrm{if}\;M\neq 0 \end{array}$$

(12) V=M

### 2.2. Image Hue Histogram

**Figure 2:**
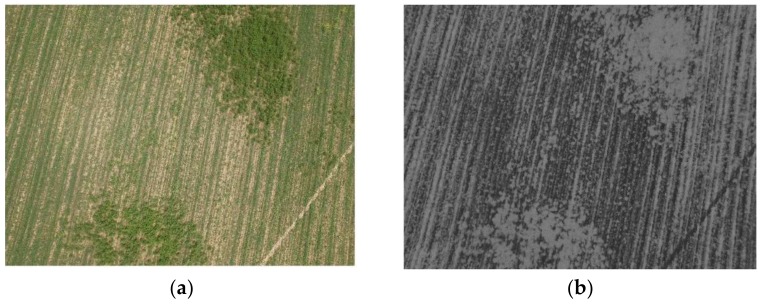
(**a**) RGB image for organic-beans field; (**b**) hue image for the same field.

After converting the image’s color space into the HSV, the hue (H) image is used to detect the suitable threshold value to discriminate between vegetation and non-vegetation objects. As discussed, the hue image provides a separate description for the color content of the objects except from the illuminance state. So, Hue image as shown in Figure 2, provides a suitable grayscale image that can be used to classify objects based on the color content. Therefore, the following step of the proposed vegetation segmentation technique is the threshold detection using the Hue image and its histogram.

**Figure 3:**
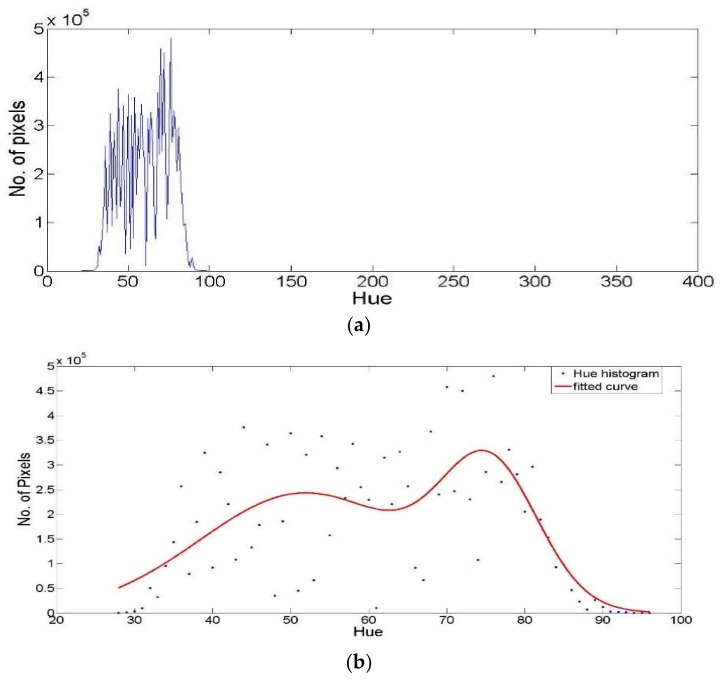
(**a**) Hue histogram; (**b**) the fitted Gaussian curve of the hue histogram.

Generally, a histogram of a hue image represents a relationship between each hue value and the number of pixels that have the same hue value, as shown in Figure 3. The use of image histogram for threshold detection was introduced by different techniques [27,32,36]. These techniques generally were implemented on vegetation segmentation process with the assumption that there are two dominant classes, the vegetation and the non-vegetation, where these classes’ colors are distributed following the normal or Gaussian distribution. Based on the probability theory, the threshold value that discriminates between these two classes represents the value that discriminates between the two Gaussian distributions. Such concept is followed by many different threshold index-based techniques as well as the proposed methodology.

**Figure 4:**
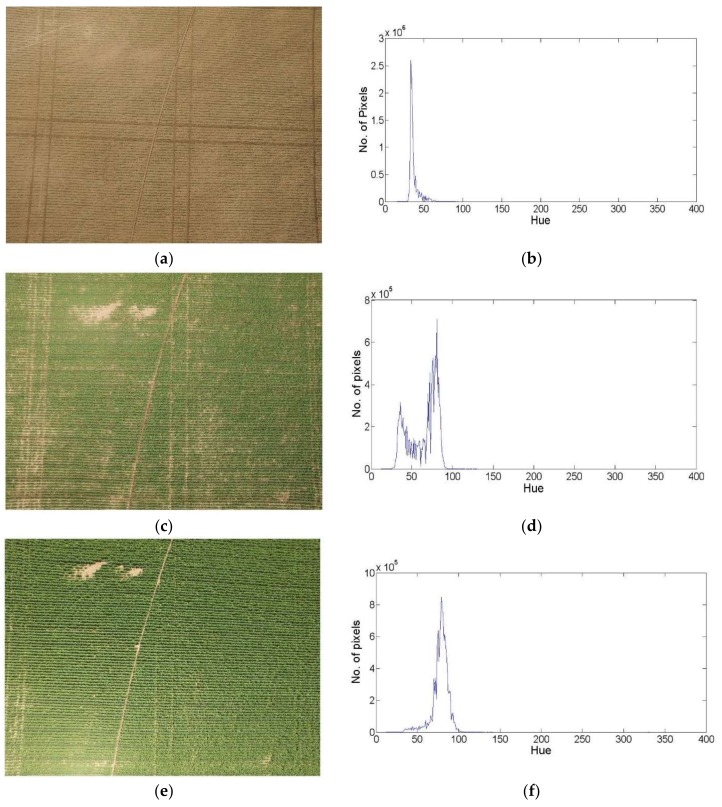
RGB images and hue histogram for canola crop at three different growth stages. (**a**,**c**,**e**) represent RGB images at 17, 29, 37 days after plant emerging; (**b**,**d**,**f**) are the hue histogram for these images.

As shown in Figure 4, three different images for a canola crop field at three different growth stages are presented. These images show that the existence of the vegetation and non-vegetation classes is not equal through the different growth stages, as can be shown within the Hue histogram for each image. Moreover, the color distribution of one class, either vegetation or non-vegetation, might be significantly unnoticeable in the image. Thus, following the assumption that the color of these two classes will be distributed with two separate Gaussian distribution is not always valid.

Therefore, the proposed threshold detection technique assumes that there will be at least one main class, either vegetation or non-vegetation, that its color content is distributed following Gaussian distribution. Through such assumption, the proposed threshold detection process is based on detecting the limits of the main Gaussian distribution in the image histogram. The values of these limits or borders represent the threshold values that can discriminate the dominant class, either the vegetation class or the non-vegetation class, from the other class in the image. To detect that suitable threshold, two different graphs are generated from the hue histogram of the collected field image, to discriminate the dominant class hue color distribution. These graphs are a filtered hue histogram and a fitted Gaussian curve for the hue histogram, as shown in Figure 3.

On step two, the proposed vegetation technique filters the hue image histogram to remove any hue value that has small presence in the image. Such filtration process is performed based on the number of pixels for each hue. So, if any hue value is represented with less than 0.001% of the number of image’s pixels, the proposed process classifies it as a small presence hue, and therefore remove this hue value from the hue histogram. The main motivation for such step is to work as an outlier remover procedure that remove low importance hue values.

Then, the following step is to fit the filtered hue histogram with a normal distribution, or Gaussian curve. The basic concept for curve fitting is to find the fitting parameters (a,b,c), of Gaussian Equation (13) using the hue histogram (x,y) data. For such purpose, different approaches can be used to estimate these parameters as Gauss-Newton algorithm, or its modification, the Levenberg-Marquardt algorithm [37].

(13) $$y=\overset{n}{\underset{i=1}{\sum }}a_{i}\times e^{\left[-{\left(\frac{x-b_{i}}{c_{i}}\right)}^{2}\right]}$$

where a is the amplitude, b is the centroid, c is related to peak width, and n is the targeted number of peaks. As the main assumption is the existence of two main Gaussian distribution, that represents the two main classes, therefore the number of terms (n) is selected to be 2. 

### 2.3. Threshold Detection Process

**Figure 5:**
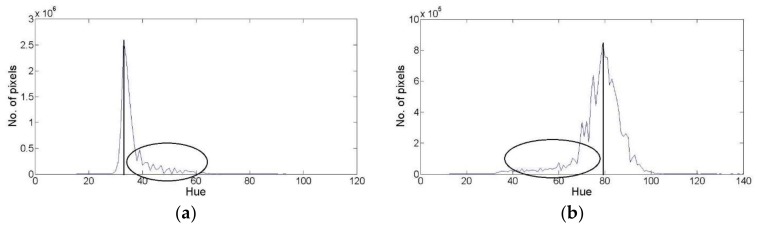
Location of possible thresholds (marked within ellipses). (**a**) the case of non-vegetation is the dominant class; (**b**) the case of vegetation is the dominant class.

The third step of the proposed vegetation segmentation technique aims to detect the suitable threshold value. As explained, the proposed vegetation technique assumes that the threshold value exists beyond the limits or the boarders of the dominant class’s color distribution, as shown in Figure 5. Therefore, the proposed technique introduces an alternative to detect different possible threshold values to produce the final suitable threshold for the image binarization process. These values aim to discriminate the dominant class’s colors from the remaining colors. So, the generated two graphs of hue histogram and fitted Gaussian curve are used to detect these values. The following parts explain these steps.

The threshold detection process starts with detecting the dominant hue value in the dominant class. This hue value can be detected as the mean of the dominant class Gaussian curve (mean_dominant) or the highest represented hue value from the filtered hue histogram (main_Hue). Such value is important for classifying the dominant class as vegetation or non-vegetation, and for detecting the direction of searching for the threshold values, either to the right or the left of the mean value, as shown in Figure 5. The (mean_dominant) is used for the classification process of the dominant class using 60° as a threshold value to discriminate between vegetation and non-vegetation. Such threshold value is accepted to be used for the classification of the mean value of the dominant class. Such value was chosen as the green color hue value, which is the main color of vegetation objects, ranges from yellow color at 60° till cyan color at 180° [38]. Such range can be used to classify the main Hue value in the dominant class, but won’t be suitable to classify each hue value. This is caused as the edge that filters between yellow and green is affected with different factors, therefore using a specific value to discriminate between yellow and green is not valid for all images. Therefore, the suggested range can be used to classify the main hue value, but can’t be used to classify all the values.

After classifying the dominant class, or color, in the hue image, the threshold searching direction can be detected. Also, through detecting the number of peaks, or the Gaussian curves in the fitted Gaussian graph, the system classifies the image case into four main types. These case types are:

- Case (1): the image has one Gaussian peak and the dominant class is non-vegetation.
- Case (2): the image has one Gaussian peak and the dominant class is vegetation.
- Case (3): the image has more than one Gaussian peak and the dominant class is non-vegetation.
- Case (4): the image has more than one Gaussian peak and the dominant class is vegetation.

**Figure 6:**
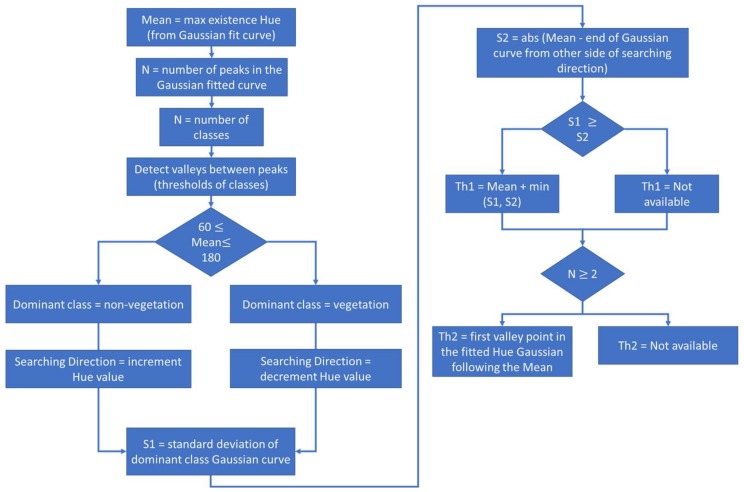
Flowchart for detecting th1 & th2.

Then, using the two hue graphs, different suitable threshold values are detected. These values propose different potentials to describe the boarders of the dominant class, where the threshold values are assumed to be the local minima within these boarders. First, the hue Gaussian fitted graph introduces two possible threshold values, which are (th_1) and (th_2). The first threshold, (th_1), represents a description for the boarders of the dominant class Gaussian curve as the sum of the (mean_dominant) value and the suitable confident interval value, between (σ, 2σ, or 3σ), of the Gaussian curve, as shown in Figure 6. It is important to note that the process neglects (th_1) if the confident interval value is equal or larger than distance (S2), which represents the difference between the end of the fitted Gaussian curve, from the other side of the threshold searching direction, and the mean Hue value (mean_dominant). Such assumption is followed as indication that the Hue histogram is fitted with Gaussian curve correctly.

Theoretically, the Gaussian curve is symmetric around its mean value. Therefore, in the hue histogram, the difference between the mean and the confident interval can’t be a negative value, as this would be an indication that the suggested Gaussian curve didn’t fit the hue histogram correctly. Thus, if the confident interval is larger than the threshold (S2), the first threshold value (th_1) is rejected. 

The second threshold value derived from the hue Gaussian fitted graph is (th_2). This value is only detected if there is more than one Gaussian curve in the hue Gaussian fitted graph, which works for cases (3) and (4). Generally, (th_2) represents the valley point between the dominant class Gaussian curve and the following Gaussian curve, based on the detected searching direction. Such point represents a potential description for the dominant class Gaussian curve boarders, or a local minima value.

**Figure 7:**
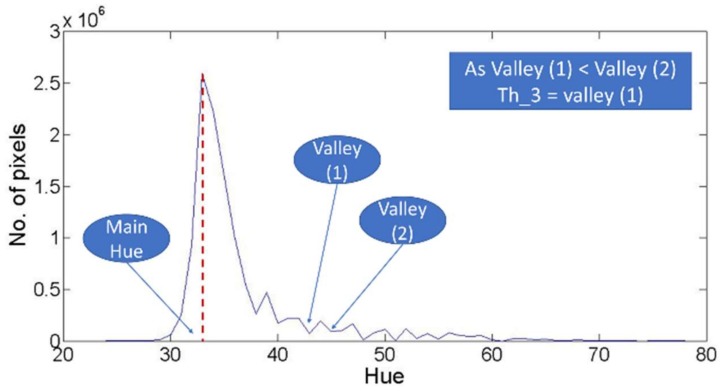
Description for th_3.

The second graph used for threshold detection is the filtered hue histogram. This graph is used to detect three potential threshold values which are (th_3), (th_4), and (th_5). These threshold values are providing three different approaches for describing the local minima. The proposed three approaches aim to detect the local minima value that discriminate the dominant color, or class, in the agriculture field image from the remaining colors. First, (th_3). represents the first valley point which is smaller than the following valley in the filtered Hue histogram, as shown in Figure 7. Such threshold value will be referred as the first lowest valley following the mean hue value. For more description, (th_3) is detected through finding the different valleys followed the main value in the hue histogram. Then, if the number of pixels in the first valley is lower than the number of pixels of the second valley, then the first valley is considered (th_3). If the described condition didn’t work, then the comparison goes for the following two valleys, till the system reaches the suitable valley for (th_3). 

**Figure 8:**
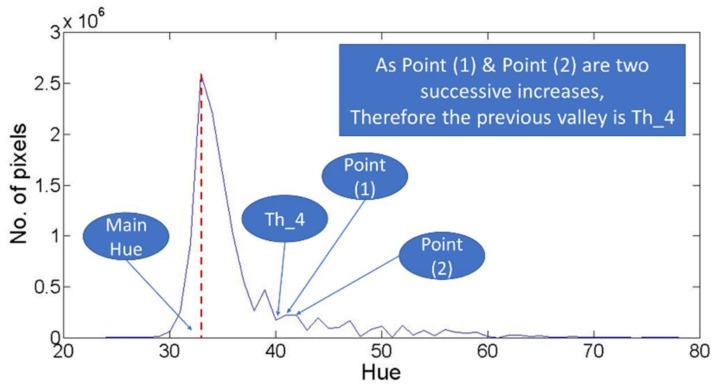
Description for th_4.

**Figure 9:**
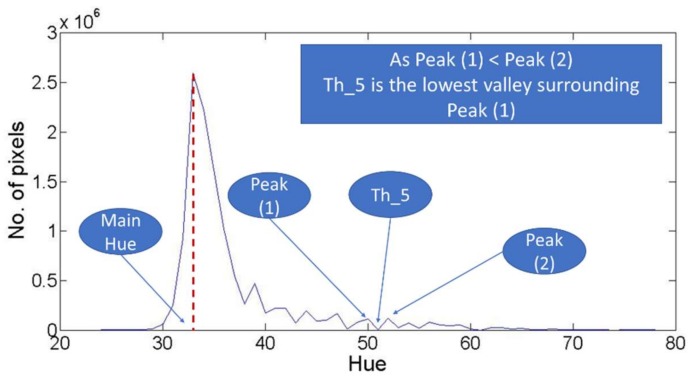
Description for th_5.

The second detected threshold value in the filtered hue histogram is (th_4) which represents the first valley point followed by two successive uprising, as shown in Figure 8. Finally, the third detected threshold is (th_5). This threshold value is determined through detecting the first peak point smaller than the following peak, based on the number of pixels. After detecting this peak, the following and previous valleys are compared. Then, the smallest valley, also based on the number of pixels, is considered as the (th_5), as shown in Figure 9. Finally, it is important to note that the existence of the five different threshold cases is not achieved in every image, as some of these thresholds cases cannot be existing in all images. Such note is mainly derived as some the described thresholds were not detected in some cases, as will be explained through the results discussion.

### 2.4. Image Vegetation Binarization

Finally, the generated thresholds values are used to generate the vegetation binary image, where the vegetation objects are presented with white color and the remaining objects are presented with black color. As the thresholds values were excluded from the Hue channel, the proposed technique applies the detected threshold value on the grayscale Hue image to generate the vegetation binary image. As the binarization process is performed using one threshold value, it was important to generate the final threshold value from the detected potential values.

Therefore, it is challenging to find the suitable threshold value within the detected different thresholds. Thus, it was important to collect different RGB images using low-cost UAV imagery system. Such images are providing different cases to test the different thresholds and evaluate their performance. Finally, based on the performance analysis of these threshold, the suitable threshold value for the proposed methodology could be detected.

## 3. Methodology Implementation

**Figure 10:**
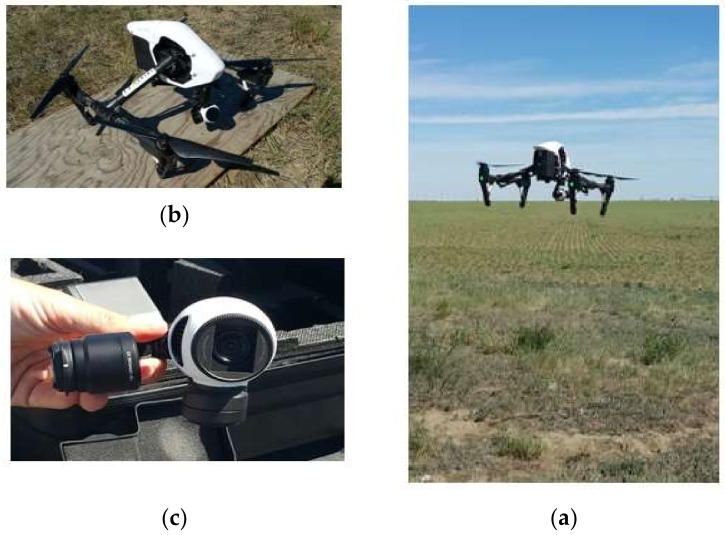
(**a**) UAV imagery system from DJI; (**b**) Inspire 1 drone; (**c**) X3 RGB camera.

As shown in Figure 10, an Inspire 1 UAV from DJI (Shenzhen, China) equipped with low-cost DJI Zenmuse X3 RGB camera was used to collect different images over three different agricultural fields located in the Scandia (AB, Canada) area. Table 1 summarizes the specs of the used X3 RGB camera. The targeted crops were canola, soybean, and organic beans. The collected images were used to evaluate the proposed methodology. The collected images for the three different fields, where collected at two or three different growth stages. The first crop, the canola, was emerged on 18 May 2017. For this crop, three data collection sessions were performed to collect the images, specifically on 3, 15 and 23 June 2017. At each session, the UAV was flown at 40, 80, and 120 m height. Using the same flying heights, the UAV was used to perform one flight sessions for the second crop which is soybeans. The soybeans emerged on 26 May 2017 and the session was on 15 June 2017. For the third crop, the organic beans, two flight sessions were performed to collect the RGB images. The sessions were on 23 June and 1 July 2017. The flying heights for the first session were 20, 80, and 120 m, while for the second session, the flying heights were 20, 40, 80, and 120 m.

The acquired RGB images present different cases of crops, illumination conditions, growth stages, and flight heights. These cases provide different scenarios which can test and evaluate the proposed methodology. The images were collected using the mentioned flight sessions, where from each session different images were selected to test the proposed methodology. The total number of the selected images are 34 images, which was divided into two main groups. The first group, contains 12 images, with focus on the low flight height images as listed in Table 2. This group of images was used to solve some challenging problem in the proposed methodology, as will be explained in the following parts. Later, the remaining 22 images, as listed in Table 3, were used to evaluate the methodology segmentation accuracy.

It is important to note that all the used images are raw images acquired from the imagery sensor without any modification or preprocessing. Moreover, for clarification, the different growth stages expression is used as an indication to the change of the vegetation canopy or the vegetation density in the field. Generally, all the collected images were at the vegetative phenological stage. Finally, the proposed methodology was tested using the acquired images individually instead of generating the mosaic of the image and then use it to test the methodology. The main reason for such orientation was mainly to make the use of UAV imagery systems for PA applications more computationally efficient.

## 4. Experimental Results

As discussed, the proposed methodology aims to detect a suitable threshold that can be used for the vegetation binarization process. The proposed procedure detects five different thresholds using two graphs driven from the hue image. These thresholds can be used to generate the vegetation binary images as the main output for the vegetation segmentation process, so it is important to generate ground truth vegetation binary images to be used for accuracy evaluation of the binarization and threshold detection.

The ground truth binary images were generated using an image manipulating program called GIMP, which is a free and open-source graphics editor software. Generally, GIMP, same as other image manipulating programs, provides the user with the ability to convert the image’s colors. So, for each RGB image, the vegetation objects were manually selected, and their colors were changed to be white while the remaining colors were converted to be black. Finally, for quantifying the vegetation binarization process accuracy, each binary image, generated with the proposed methodology, was pixel by pixel compared to its ground truth binary image generated with GIMP. But first, it is important to solve few challenges that we face when implementing the proposed methodology.

First, the threshold detection process provides five thresholds which can provide different values, so it is important to detect the suitable threshold value that achieve a sufficient binarization accuracy. Second, as discussed, th_1. depends on computing the confident interval of the dominant Gaussian distribution of the dominant hue color, so it is important to check the suitable confident interval value between (σ, 2σ, or 3σ), which achieves the highest binarization process accuracy.

The third challenge is related to th_3, th_4, and th_5. These three thresholds aim to detect the local minima point as an indication for the end of the dominant hue color distribution in the hue histogram. The challenge for these thresholds is caused by the fluctuations within the hue histogram. Such fluctuations could easily cause a wrong selection for the threshold point, so for each one of these three thresholds, the vegetation binarization process was performed using different values. These values represent the different successive selected points that fit the description of the threshold. For more explanation, th_3 is considered as the first lowest valley as described in Figure 7. So, is it suitable to detect the first lowest valley and stop? Or should the system continue for the second lowest valley? Or should the system continue for successive possible points? The challenge here is to find the suitable stop for searching th_3 and similarly th_4 and th_5. Therefore, based on the computed accuracy derived from each of different possible values for each threshold case, such challenging questions could be answered.

As the first challenge, which search for the suitable threshold value between the proposed five thresholds, it is important to solve any challenges related to each one of them. Therefore, the following part works on solving the second and third challenges first. Thus, 12 different RGB images from the described flight sessions were used. These images, as listed in Table 2, provide different crops, flight height, growth stages, and illumination conditions. For selecting the suitable confident interval, which is related to th_1 computation, each image was binarized using the detected th_1 only using one of the three different values of confident intervals, which are σ, 2σ, and 3σ, for each case. The achieved accuracies were then listed in Table 4.

Similarly, for solving the third challenge, which is related to th_3, th_4, and th_5, each one of them is evaluated seperately. As it is important to detect the suitable stop point for searching the local minima for each threshold, so the accuracy of the binarization process for each detect local minima is computed. These accuracies for the different 12 RGB images are stated in Table 5, Table 6 and Table 7. The main motivation for such results is to find the suitable stop of each threshold.

The following parts are providing an analysis for the presented results through the previous tables. Such analysis is targeted to solve the mentioned challenges. Then, the main challenge of choosing the suitable threshold value form the proposed five thresholds, can also be solved through comparing the accuracy performance of each threshold. Moreover, the proposed methodology was compared with three other color-index based approaches which are the ExG, ExGR, and NGRDI. These three vegetation indexes are used to generate a suitable grayscale image from the input RGB image. Then, using the automatic Otsu threshold detection, the grayscale image is binarized. Also, the implementation of Otsu threshold detection is used for the Hue image as the fourth approach. Finally, the accuracies of these four approaches were used to evaluate the performance of the proposed methodology compared to these well-known approaches (i.e., ExG + Otsu, ExGR + Otsu, NGRDI + Otsu, and Hue + Otsu). These approaches present accurate segmentation process based on their performance mentioned in surveying researches [19].

## 5. Results Analysis

In this section, a discussion is provided for the acquired results, as shown in Table 3, Table 4, Table 5 and Table 6, to solve the mentioned challenges related to the selection of suitable confident interval of the standard deviation for th_1 and the suitable stop for detecting th_3, th_4, and th_5. Later, through comparing the performance of the five different proposed thresholds, the suitable threshold value for the vegetation binarization can be derived. Finally, an analysis for the performance of the proposed methodology is provided through comparing the segmentation accuracies of the proposed vegetation segmentation methodology and four other accurate methodologies.

### 5.1. Confidence Interval of the Dominant Gaussian

**Figure 11:**
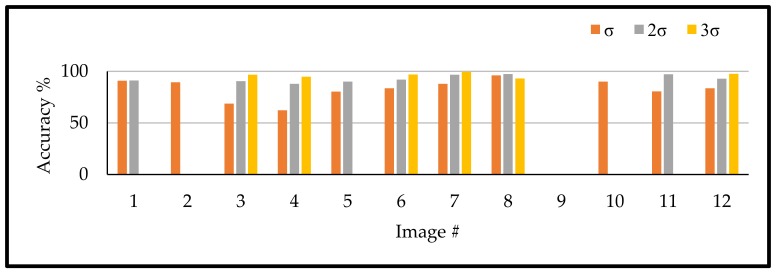
Binarization accuracies for different confident intervals.

As discussed, detecting the suitable confident interval is a challenging problem for detecting the suitable th_1. So, it was important to compare between the different intervals of σ, 2σ, and 3σ for *th*_1. Based on the achieved accuracies, as shown in Table 4 and Figure 11, generally, as the confident interval increases, the binarization accuracy also increases. The majority of the tested images showed higher binarization accuracy with the use of the higher allowable confident interval. The only exception was for image 8 where the 2σ confident interval showed higher accuracy. Thus, the most suitable solution for selecting the confident interval for th_1 is using the highest allowable confident interval. Finally, it is worth to mention that some of the confident intervals failed to follow the discussed (S2) condition. Therefore, some of the images didn’t have a threshold at every confident interval and even more at image (9) all the confidence intervals thresholds were failed.

### 5.2. Local Minima Searching Limit 

In the proposed methodology, the selection of a suitable local minima as a threshold to discriminate the dominant color histogram, is considered a challenging problem. Therefore, Table 4, Table 5 and Table 6 are providing the achieved accuracies for different local minima points that fit the description for th_3, th_4, and th_5. These tables were used to find an approach for selecting the suitable stop limit for searching for these thresholds.

For th_3, the achieved accuracies in Table 5 shows that there is no clear trend for the different stops’ accuracies to select the suitable stop searching limit. Through the listed accuracies, it was obvious that in some cases as in images 1, 2, 3, or 4, the first detected stop for th_3 provides the highest accuracy, while in remaining images the highest accuracy was for middle stops. Therefore, computing the average as the suitable threshold might be considered as the reasonable solution based on the achieved accuracies.

**Figure 12:**
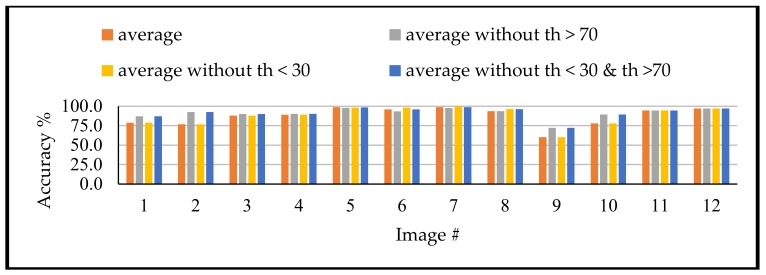
Achieved accuracy for different modification for th_3.

Moreover, the achieved accuracies showed that the thresholds values larger than 70° and the thresholds less than 30° are providing lower accuracies compared to the remaining values. Therefore, for detecting the suitable th_3, the methodology should remove any threshold higher than 70° or less than 30°. To that end, the suitable th_3 is the average of the detected values that fit the th_3. description after removing any value larger than 70° or less than 30°. Such approach was approved, as shown for the different tested images through Figure 12, which compare the achieved accuracies for computing th_3 through four different approaches. The first approach is the average of all detected th_3. points. The second is to average all stops after removing the values larger than 70°. The third approach is to average of all stops after removing the values less than 30°, while the last approach is the average of all stops after removing the values less than 30° and larger than 70°.

**Figure 13:**
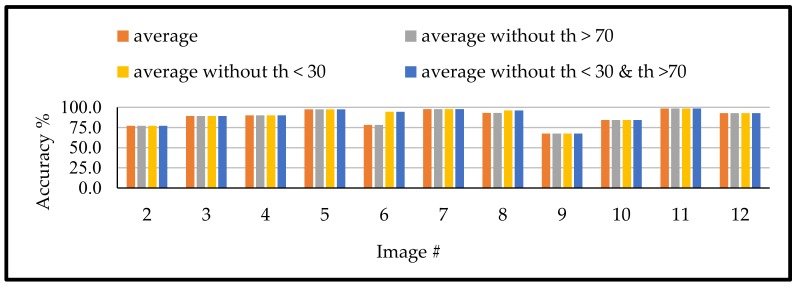
Achieved accuracy for different modification for th_4.

**Figure 14:**
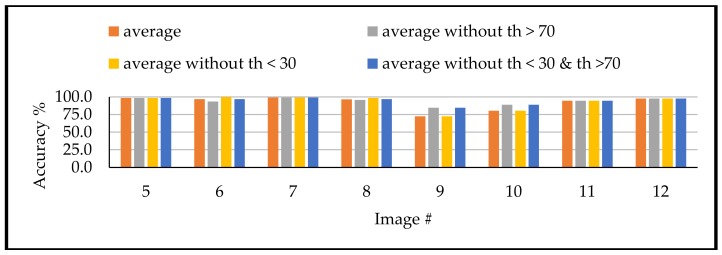
Achieved accuracy for different modification for th_5.

Similarly, for th_4 and th_5. accuracies, Table 6 and Table 7 showed that the threshold values larger than 30° and less than 70° are providing the highest accuracies. Such conclusion can be derived through Figure 13 and Figure 14. These figures show that the binarization accuracy for any image is higher when removing the values larger than 70° and less than 30° from the detected thresholds.

Finally, the previous analysis for the different cases accuracies for different thresholds, proposed a suitable solution for each challenge for th_1, th_3, th_4, and th_5. Such solutions are used for the main challenge to evaluate each threshold from the proposed 5 thresholds. So, each threshold is used to generate the binary image and through the ground truth images, the threshold binarization accuracy is generated.

### 5.3. Final Threshold Value

The main target of the proposed methodology is to generate a vegetation binary image. Such image, in the proposed methodology, is generated using the hue image and a suitable threshold value. As discussed, the methodology introduces five different threshold values from the fitted Gaussian graph and from the filtered hue histogram. Therefore, it is important to detect the suitable threshold value from these five candidate thresholds. As shown in Table 8, there is no clear threshold provides the best accuracy performance within the five different thresholds in all cases. Generally, th_1. is showing the highest accuracy, but it is not detected in all cases. Also, th_3 is detected in all cases, but it didn’t provide the highest accuracy. Moreover, as shown in Table 8, it is important to note that some of the thresholds failed to be detected in some cases as can be seen in images (1, 2, 3, 4, 7, 8 and 9). Thus, the average of these threshold values would be considered as a satisfied solution for the suitable threshold value.

### 5.4. Proposed Methodology Performance Evaluation

As mentioned, different researchers have developed different vegetation segmentation methodologies that can be used for different agriculture applications as weed detection. These researchers use different accuracy evaluation approaches. Generally, these accuracy evaluation approaches depend on using an image as a benchmark. Then the vegetation segmentation accuracy is evaluated through comparing the generated binary image with the benchmark. Finally, the segmentation accuracy is measured through the ratio of correct classification of pixels.

The mentioned benchmark is generated through different techniques. For example, different researchers manually generated the benchmark image using Photoshop [7,39]. Other researchers generated the benchmark image using other vegetation segmentation methodologies [25]. Moreover, a review article for the vegetation extraction and segmentation techniques performed a dependent evaluation for different vegetation segmentation techniques through generating a benchmark image using Photoshop [19]. Therefore, the proposed vegetation segmentation methodology was evaluated using a similar process through generating a benchmark image using GIMP software which is free software similar to Photoshop.

In this subsection, an evaluation of the proposed methodology segmentation performance is provided through comparing the achieved accuracy for the binarization process between the proposed methodology and other well-known approaches. As mentioned, these methods are generally considered as color index-based approaches which generate a special grayscale images from the inputted RGB images. Then, using Otsu threshold detection, the vegetation binary images are generated. The used color index methods are ExG, ExGR, NGRDI, and Hue, which were selected based on their accurate performance [19]. This comparison was performed using the two groups of images as described in Table 2 and Table 3.

**Figure 15:**
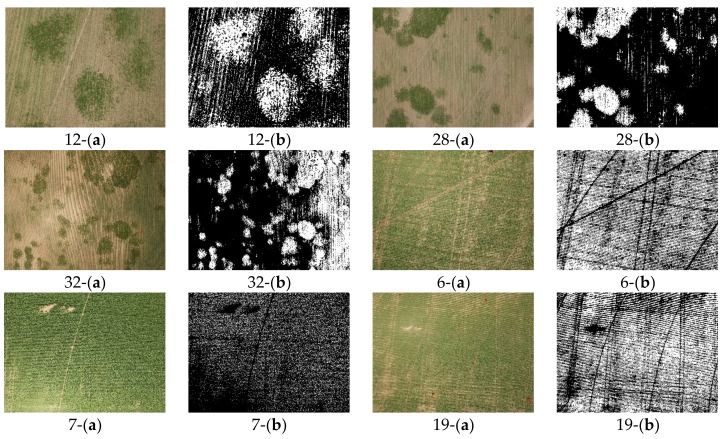

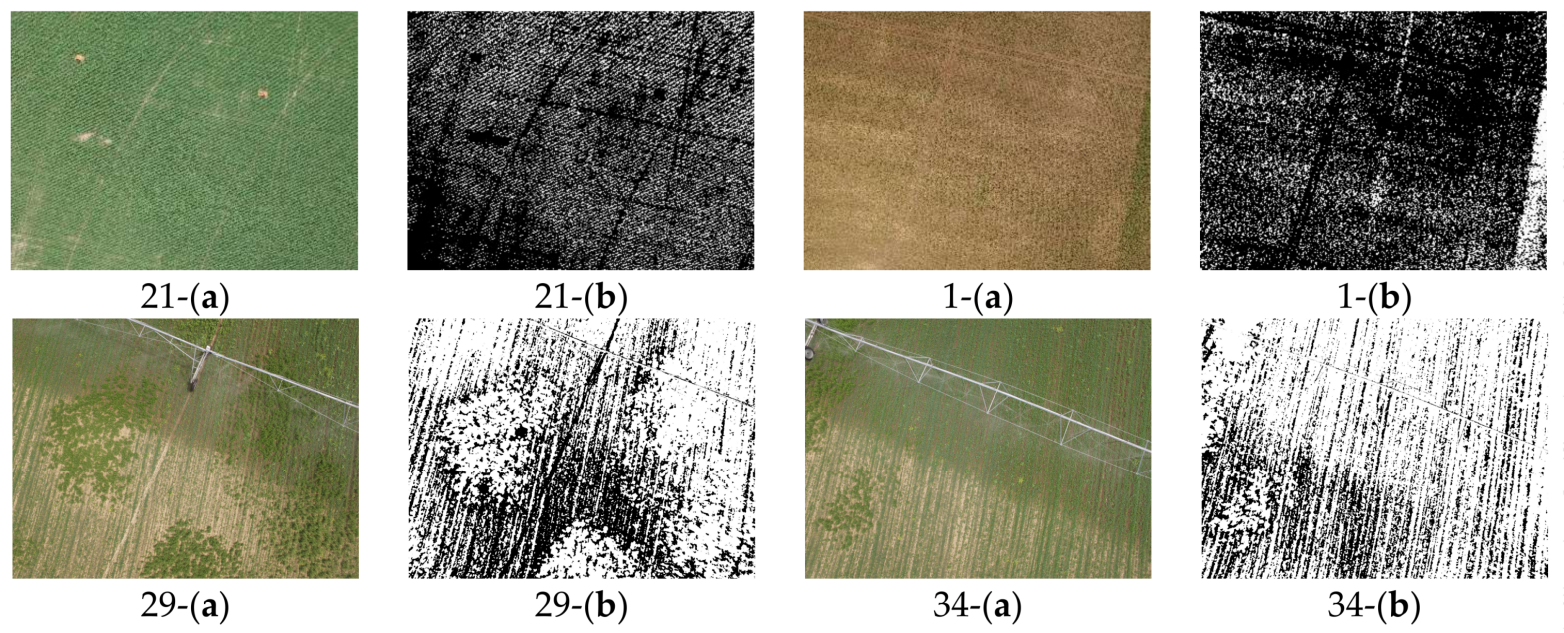
Generated vegetation binary images from the proposed methodology. Each image is labeled with the image number, while (**a**) is for the acquired RGB image, and (**b**) for the generated binary image.

**Figure 16:**
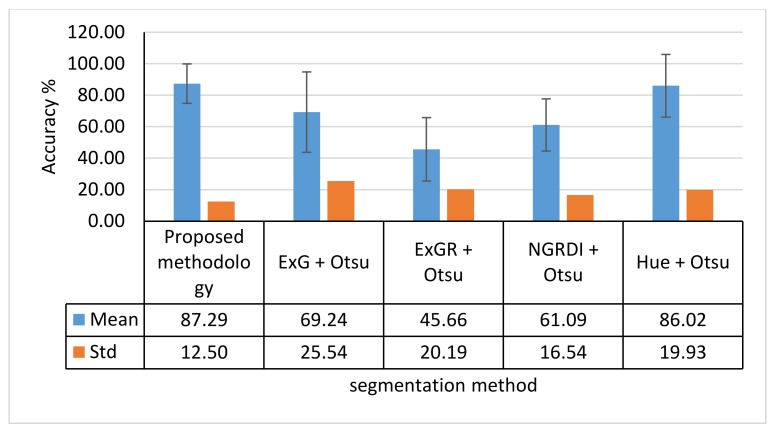
Performance comparison between the proposed methodology and other approaches. Std is represented by error bar.

These different 34 RGB images, were used to evaluate the proposed methodology along with the other mentioned ExG + Otsu, ExGR + Otsu, NGRDI + Otsu, and Hue + Otsu methods. The achieved results from segmenting the 34 RGB images showed a reasonable stability and consistency binarization performance, as shown in Figure 15. Such performance stability is evaluated through comparing the mean and standard deviations of the proposed method to other methods as shown on Figure 16.

The acquired accuracies showed stable and accurate segmentation process, even with the changes of illumination conditions, crop type, or growth stage. The stability of segmentation process was assessed using the standard deviation of the accuracies for the different 34 RGB images. However, the low segmentation accuracies were generally caused by the low resolution of the collected RGB images. Such low-resolution effect can be noticed through comparing the achieved accuracies when changing the flight height for the same crop and same growth stage, as shown for the achieved binarization of images (29), (30), (31), and (32) in Table 9.

## 6. Conclusions

The proposed paper introduced a new vegetation segmentation approach which aims to generate vegetation binary images from RGB images acquired by a low-cost UAV imagery system. Such vegetation binary images can be used for weed management, crop row detection, and crop health monitoring applications, which depend on discriminating the vegetation from non-vegetation objects as an initial step for the application. Generally, the proposed approach depends on detecting a suitable threshold value using the hue histogram of the RGB image. Then, the detected threshold value, is used to perform a binarization process for the grayscale hue image and the generated binary image is considered as an output for the vegetation segmentation process. Later, to evaluate the proposed methodology, different UAV flight sessions for different agriculture fields were performed to collect different RGB images. These images, as explained, provided variety of cases that can evaluate the vegetation segmentation performance of the proposed methodology.

Based on the achieved results, the proposed methodology was capable to generate vegetation binary images that discriminate the vegetation objects from non-vegetation objects for different cases. These cases involved different flight heights, illumination conditions, crop types, and growth stages. The mean accuracy for the segmentation process for all the tested images reached 87.29% with standard deviation equal to 12.5%. The main advantage of the proposed methodology is its ability to successfully detect vegetation objects regardless the flight height and illumination conditions. Such ability can be shown in the stability of the segmentation accuracy based on the error standard deviation, while other well-known approaches such as ExG + Otsu, ExGR + Otsu, and NGRDI + Otsu showed different standard deviation ranges from 16.54 to 25.54 as shown in Figure 16.

Moreover, it is recommended to use the proposed methodology for segmenting the collected images for flight height 40 m or less, as the higher heights images were highly affected with the resolution of the used camera. Such recommendation is preferred if the targeted PA application is crop row detection or crop health monitoring. On the other hand, if the targeted application is weed management, the achieved results from 120 m flying height images showed the ability to represent the weed spots, as shown for the achieved segmentation accuracies in Table 9 for images (19), (21), and (32). Therefore, implementing such segmentation approach can be easily used to develop different weed patch detection and weed management applications. Such recommendation is based on the ability of the proposed segmentation approach to handle RGB images acquired at high altitude, which provide the ability to cover the agriculture filed faster and with less number of images compared to current UAV weed management techniques that recommend to collect the images at 40 m [16]. 

Finally, it is important to note that the proposed method provided the highest segmentation accuracy compared to the hue + Otsu approach as shown in Figure 16. However, the main reason that the general accuracy of the proposed methodology was better that the hue + Otsu approach is due to the low accuracy achieved at three particular images, which are images 29, 33, and 34. This is mainly due to the special situation of the field. These images were taken for organic bean field while it was irrigated. Therefore, the color spectral of these images were different from other images for the same field.

Therefore, the proposed vegetation segmentation methodology can be considered as a stable methodology especially as it provided the highest segmentation accuracy in many different cases, as shown in images 18, 19, 21, 22, 24, 25, 29, 33 and 34. Also, the proposed method least segmentation accuracy is 53.73%, while the hue + Otsu least segmentation accuracy is 16.74%, based on the achieved results summarized in Table 9.

## Figures and Tables

**Table 1:** DJI RGB X3 sensor specs.

Camera Specs	
Sensor Size	6.17 × 4.55 mm
Operating Temperature	0° to 40° C
Focal length	~4 mm
Image Format (pixels)	4000 × 3000
Sensor type	Sony EXMOR CMOS

**Table 2:** List of group 1 of images used for solving the methodology challenges.

Image #	Crop	Flight Height (m)	Date	GSD (cm)
(1)	Soybean	40	15 June	~1.54
(2)	Soybean	40	15 June	~1.54
(3)	Canola	40	3 June	~1.54
(4)	Canola	40	3 June	~1.54
(5)	Canola	40	15 June	~1.54
(6)	Canola	40	15 June	~1.54
(7)	Canola	40	23 June	~1.54
(8)	Canola	40	23 June	~1.54
(9)	Organic Bean	20	23 June	~0.77
(10)	Organic Bean	20	23 June	~0.77
(11)	Organic Bean	20	1 July	~0.77
(12)	Organic Bean	20	1 July	~0.77

**Table 3:** List of group 2 of images used for evaluating the methodology.

Image #	Crop	Flight Height (m)	Date	GSD (cm)
(13)	Soybean	40	15 June	~1.54
(14)	Soybean	40	15 June	~1.54
(15)	Soybean	80	15 June	~3.1
(16)	Soybean	120	15 June	~4.63
(17)	Canola	40	3 June	~1.54
(18)	Canola	40	15 June	~1.54
(19)	Canola	80	15 June	~3.1
(20)	Canola	80	15 June	~3.1
(21)	Canola	120	15 June	~4.63
(22)	Canola	40	23 June	~1.54
(23)	Canola	40	23 June	~1.54
(24)	Canola	80	23 June	~3.1
(25)	Canola	80	23 June	~3.1
(26)	Canola	120	23 June	~4.63
(27)	Organic Bean	20	23 June	~0.77
(28)	Organic Bean	80	23 June	~3.1
(29)	Organic Bean	20	1 July	~0.77
(30)	Organic Bean	40	1 July	~1.54
(31)	Organic Bean	80	1 July	~3.1
(32)	Organic Bean	120	1 July	~4.63
(33)	Organic Bean	20	1 July	~0.77
(34)	Organic Bean	20	1 July	~0.77

**Table 4:** Binarization accuracies for different confident intervals.

Image #	Accuracy %		
	σ	2σ	3σ
(1)	90.9	91.1	N/A
(2)	89.4	N/A	N/A
(3)	68.7	90.4	96.7
(4)	62.2	88.0	94.9
(5)	80.4	90.0	N/A
(6)	83.6	92.1	97.0
(7)	87.8	96.8	99.5
(8)	96.1	97.3	93.2
(9)	N/A	N/A	N/A
(10)	90.1	N/A	N/A
(11)	80.6	97.1	N/A
(12)	83.6	92.9	97.7

**Table 5:** Binarization accuracies for different stops for th_3 .

Image #	th_3/Accuracy %										
	1	2	3	4	5	6	7	8	9	10	11
(1)	45	73									
	87.2	78.2									
(2)	45	73									
	92.5	75.4									
(3)	43	51	61	74							
	95.2	89.9	87.5	87.1							
(4)	43	51	55	61	74						
	96.6	91.0	89.7	88.4	87.8						
(5)	73	67	61	55	51	48	45	43	41	25	
	75.1	86.6	91.6	97.0	98.7	97.5	94.7	92.5	89.6	71.6	
(6)	73	67	61	55	51	48	45	43	41	38	21
	78.2	89.8	94.9	99.8	96.7	94.6	91.8	89.7	86.7	80.9	60.1
(7)	73	61	51	43	28						
	85.0	98.3	98.6	97.1	95.5						
(8)	61	57	51	45	43	41	25	13			
	98.7	98.8	96.6	94.8	94.1	93.5	90.8	90.7			
(9)	43	51	55	61	67	74	78	82			
	99.9	79.2	72.3	62.5	57.1	51.5	48.9	47.4			
(10)	43	48	51	55	61	67	74	78	82		
	84.3	98.5	95.8	89.2	79.3	72.3	62.6	57.3	53.8		
(11)	66	61	48	45	33						
	75.4	85.5	89.6	84.4	65.0						
(12)	67	61	55	48	43	40	38				
	76.7	87.1	94.9	93.0	84.9	79.1	74.4				

**Table 6:** Binarization accuracies for different stops for th_4.

Image #	th_4/Accuracy %									
	1	2	3	4	5	6	7	8	9	10
(1)	58									
	77.1									
(2)	45	48	66							
	93.4	91.1	87.2							
(3)	45	48	66							
	94.7	92.2	87.9							
(4)	77	76	68	58	50	25	24	14	13	7
	43.9	44.8	53.5	65.2	80.6	78.7	78.7	78.6	78.6	78.6
(5)	47	55	58	67	68	73				
	67.5	61.3	59.2	54.7	54.6	52.4				
(6)	68	51	41	38	37					
	94.7	98.6	96.7	96.2	96.1					
(7)	68	51	50	41	38	23	22	21		
	94.0	96.6	96.3	93.5	92.5	90.8	90.8	90.8		
(8)	45	48	57	61	67	68				
	93.6	84.6	69.5	62.5	57.1	57.1				
(9)	45	48	61	67	68					
	90.5	98.5	79.3	72.3	72.3					
(10)	66	43								
	75.4	80.1								
(11)	58	51	43	38						
	92.9	97.0	84.9	74.4						
(12)	58									
	77.1									

**Table 7:** Binarization accuracies for different stops for th_5.

Image #	th_5/Accuracy %								
	1	2	3	4	5	6	7	8	9
(5)	67	61	55	55	48	48	43	40	
	86.6	91.6	97.0	97.0	97.5	97.5	92.5	88.1	
(6)	78	67	55	55	48	48	43	40	19
	64.3	89.8	99.8	99.8	94.6	94.6	89.7	85.1	60.1
(7)	61	53	50	45					
	98.3	99.1	98.4	97.5					
(8)	71	63	61	53	45	38	25		
	88.6	97.8	98.7	97.3	94.8	92.5	90.8		
(9)	40	51	55	71					
	89.8	79.2	72.3	53.8					
(10)	40	62	66	71					
	75.1	79.3	72.9	66.8					
(11)	61	55	51	48	40				
	85.5	98.8	94.6	89.6	75.1				
(12)	61	55	55	48	48	40			
	87.1	94.9	94.9	93.0	93.0	79.1			

**Table 8:** Binarization accuracies for different threshold types.

Image #	Threshold Accuracy %				
	*th*_1	*th*_2	*th*_3	*th*_4	*th*_5
(1)	91.1	N/A	87.2	0.0	N/A
(2)	89.4	N/A	92.5	77.1	N/A
(3)	96.7	N/A	89.9	89.3	N/A
(4)	94.9	N/A	90.3	90.3	N/A
(5)	90.0	91.6	98.4	97.5	98.5
(6)	97.0	97.1	96.0	94.6	96.7
(7)	99.5	N/A	98.8	97.8	99.1
(8)	93.2	N/A	96.3	96.3	96.8
(9)	N/A	N/A	72.1	67.6	84.5
(10)	92.4	91.7	89.2	84.5	88.9
(11)	97.1	89.7	94.6	98.8	94.6
(12)	97.7	79.4	97.0	93.0	97.7

**Table 9:** Binarization accuracy for the proposed methodology compared to other accurate approaches.

Image #	Vegetation Segmentation Accuracy %				
	Proposed Methodology	ExG + Otsu	ExGR + Otsu	NGRDI + Otsu	Hue + Otsu
(13)	65.70	85.95	64.78	64.76	98.70
(14)	73.82	95.71	67.60	67.93	97.71
(15)	54.86	79.66	54.55	54.55	90.84
(16)	53.73	83.47	53.45	53.45	91.62
(17)	91.10	36.93	73.36	77.83	84.71
(18)	97.16	86.83	37.52	76.08	93.72
(19)	88.45	77.00	25.37	61.78	85.13
(20)	70.84	86.38	42.18	70.67	92.19
(21)	91.55	79.91	32.78	53.40	84.02
(22)	99.53	22.44	20.28	26.38	93.87
(23)	96.02	24.64	23.80	26.44	97.26
(24)	98.79	26.80	43.03	44.84	94.73
(25)	98.28	21.10	35.12	45.21	74.25
(26)	97.84	24.32	14.67	41.12	99.39
(27)	88.72	92.31	64.61	72.17	98.58
(28)	80.30	79.50	61.97	72.62	86.57
(29)	95.40	70.51	31.64	62.63	30.64
(30)	69.31	70.80	41.48	57.73	84.38
(31)	75.21	77.12	40.14	51.10	87.30
(32)	88.16	90.36	61.75	64.50	95.80
(33)	86.92	57.01	18.28	53.22	16.74
(34)	95.59	70.12	29.64	65.50	27.65
